# Heat Stress in Dairy Cattle Alters Lipid Composition of Milk

**DOI:** 10.1038/s41598-017-01120-9

**Published:** 2017-04-19

**Authors:** Z. Liu, V. Ezernieks, J. Wang, N. Wanni Arachchillage, J. B. Garner, W. J. Wales, B. G. Cocks, S. Rochfort

**Affiliations:** 1 Biosciences Research, Agriculture Victoria, AgriBio, 5 Ring Road, Bundoora, Victoria 3083 Australia; 2 Farming Systems Research, Agriculture Victoria, Ellinbank Centre, 1301 Hazeldean Rd, Ellinbank, Victoria 3821 Australia; 3 School of Applied Systems Biology, La Trobe University, Bundoora, Victoria, 3083 Australia

## Abstract

Heat stress, potentially affecting both the health of animals and the yield and composition of milk, occurs frequently in tropical, sub-tropical and temperate regions. A simulated acute heat stress experiment was conducted in controlled-climate chambers and milk samples collected before, during and after the heat challenge. Milk lipid composition, surveyed using LC-MS, showed significant changes in triacylglycerol (TAG) and polar lipid profiles. Heat stress (temperature-humidity index up to 84) was associated with a reduction in TAG groups containing short- and medium-chain fatty acids and a concomitant increase in those containing long-chain fatty acids. The abundance of five polar lipid classes including phosphatidylethanolamine, phosphatidylserine, phosphatidylcholine, lysophosphatidylcholine and glucosylceramide, was found to be significantly reduced during heat stress. Lysophosphatidylcholine, showing the greatest reduction in concentration, also displayed a differential response between heat tolerant and heat susceptible cows during heat stress. This phospholipid could be used as a heat stress biomarker for dairy cattle. Changes in TAG profile caused by heat stress are expected to modify the physical properties of milk fat, whereas the reduction of phospholipids may affect the nutritional value of milk. The results are discussed in relation to animal metabolism adaptation in the event of acute heat stress.

## Introduction

The imposition of heat stress on domestic production animals is commonplace in tropical, sub-tropical and temperate regions. The long term breeding of dairy cattle for increased milk production has led to higher metabolic heat generation and therefore increased susceptibility to heat stress^1, 2^. The upper critical limit of the thermoneutral zone for dairy cattle is between 25 °C and 26 °C and temperature-humidity index (THI) below 72^3^. Heat stress not only affects the welfare and health of cows, but also their productivity, which in turn increases the herd management cost and reduces the profitability of dairy farms. The frequency of high-temperature days is expected to increase with climate change^4, 5^, so mitigating the negative impact of heat stress on dairy production will be an important task for the industry. Recent studies demonstrated that selection for heat-tolerant dairy cow genotypes is feasible and results in improvements in milk production and feed intake during and after heat stress events^5, 6^.

Under heat stress conditions, some physiological changes can be observed in dairy cattle, such as increased respiration rate, heart rate and core body temperature^1, 7^, but the most prominent changes in relation to animal performance are the reduction in dry matter intake and milk yield^2, 7–9^. Heat dissipation associated metabolic adaptation is an energy expensive process and is believed to be responsible for a proportion of the decline in milk production^10^.

Apart from milk yield, the composition of milk as influenced by heat stress has been investigated in numerous studies. Most studies found heat stress was associated with a decline in total protein content and total fat content^8, 11–13^. However, some authors found no significant decrease in fat percentage for cows under heat stress^14–16^.

Lipid is one of the main components of milk. The dominant fraction of milk fat is TAG (about 98%) present in the form of fat globules^17^. In addition to being an energy source, TAG composition is implicated in human health and the property of dairy products^18, 19^. The second most important fraction of milk fat is polar lipids, which are the main structural constituents of fat globule membrane and thus play a role of emulsifier ensuring the stability of milk emulsion system^20, 21^. The principal classes of milk polar lipids include phosphatidylcholine (PC), phosphatidylethanolamine (PE), phosphatidylserine (PS), phosphatidylinositol (PI), sphingomyelin (SM), lysophosphatidylcholine (LPC), lactosylceramide (LacCer) and glucosylceramide (GluCer)^18^. Besides their functional property, polar lipids in particular SM have other beneficial effects on human health. For example, SM can reduce cholesterol absorption^22^; a recent study has also demonstrated a link between SM and infant cognitive development^23^.

Despite the importance of milk lipids in human health and dairy product property, compared with total fat content, how lipid composition is affected by heat stress has attracted much less attention. Through correlation analysis between meteorological data and fatty acid (FA) traits predicted by mid-infrared spectra, it was found that the increase of THI was associated with a decrease in content of short-chain and medium-chain FA, and an increase in that of long-chain FA^12^. A similar observation was also made for milk from heat stressed cows in an earlier study^16^. However, these results were not always conclusive, since the effect of heat stress was often confounded with that of different feeding patterns across seasons. As for the effect of heat stress on TAG profile and polar lipid composition, no information was available. We hypothesised that heat stress would modify the profile of both TAG and polar lipids of milk through changing FA pool and animal metabolism. This alteration in lipid composition may have an impact on calf nutrition, human nutrition as well as the processing and quality of dairy products.

A simulated heat-stress experiment was conducted in controlled-climate chambers recently with the aim to validate the genomic selection approach in heat tolerance improvement of dairy cattle^5^. In addition to the monitoring of milk yield, dry matter intake, respiration rate and body temperature, these authors also determined the milk composition (lactose, protein and fat %) using infrared-based technique^5^. As part of a collaborative project, an aliquot of milk samples from the same experiment was brought to our laboratory and subjected to detailed lipid composition analysis.

We describe here the methodologies for detailed lipidomic analysis using LC-MS as well as the lipid composition change in heat stress conditions for both heat tolerant and heat susceptible cows; 58 most abundant TAG groups and eight classes of polar lipids were surveyed at the molecular species level.

## Results and Discussion

### Effect of heat stress on TAG profile of milk

An exhaustive LC separation combined with the use of MS/MS spectrum-based automated structural assignment software (LipidSearch) has been adopted for lipid species identification in this study. If a TAG group is defined as a series of species having the same total acyl carbon number (CN) and the same number of total double bonds (DB), 94 TAG groups comprising over 400 species (positional isomers not counted) have been identified in raw milk. Among them, 58 most abundant TAG groups were chosen for detailed study; the chemical formula, accurate mass as well as the FA composition of the main isomers (based on abundance) of these TAG groups are summarised in Table 1.

**Table 1:** List of TAG groups surveyed.

TAG group	Formula	Calculated *m/z* (M + NH_4_)^+^	Main species (isomers)		
			Species 1	Species 2	Species 3
TAG 26:0	C_29_H_54_O_6_	516.4264	4:0/10:0/12:0	4:0/8:0/14:0	8:0/8:8/10:0
TAG 28:1	C_31_H_56_O_6_	542.4421	4:0/10:1/14:0	4:0/10:0/14:1	4:0/8:0/16:1
TAG 28:0	C_31_H_58_O_6_	544.4577	4:0/10:0/14:0	4:0/8:0/16:0	6:0/10:0/12:0
TAG 30:1	C_33_H_60_O_6_	570.4734	4:0/10:1/16:0	4:0/8:0/18:1	6:0/10:0/14:1
TAG 30:0	C_33_H_62_O_6_	572.4890	6:0/8:0/16:0	6:0/10:0/14:0	10:0/10:0/10:0
TAG 32:1	C_35_H_64_O_6_	598.5047	4:0/12:0/16:1	4:0/14:0/14:1	4:0/10:0/18:1
TAG 32:0	C_35_H_66_O_6_	600.5203	4:0/10:0/18:0	6:0/8:0/18:0	4:0/12:0/16:0
TAG 34:1	C_37_H_68_O_6_	626.536	4:0/14:1/16:0	8:0/10:0/16:1	10:0/10:0/14:1
TAG 34:0	C_37_H_70_O_6_	628.5516	4:0/14:0/16:0	10:0/12:0/12:0	10:0/10:0/14:0
TAG 36:1	C_39_H_72_O_6_	654.5673	4:0/16:0/16:1	4:0/14:0/18:1	8:0/10:0/18:1
TAG 36:0	C_39_H_74_O_6_	656.5829	4:0/16:0/16:0	6:0/14:0/16:0	10:0/12:0/14:0
TAG 38:1	C_41_H_76_O_6_	682.5986	4:0/16:0/18:1	8:0/12:0/18:1	
TAG 38:0	C_41_H_78_O_6_	684.6142	6:0/16:0/16:0	4:0/16:0/18:0	8:0/14:0/16:0
TAG 40:2	C_43_H_78_O_6_	708.6143	4:0/18:0/18:1	4:0/18:0/18:2	6:0/16:0/18:2
TAG 40:1	C_43_H_80_O_6_	710.6299	6:0/16:0/18:1	8:0/14:0/18:1	10:0/12:0/18:1
TAG 40:0	C_43_H_82_O_6_	712.6455	8:0/16:0/16:0	6:0/16:0/18:0	4:0/18:0/18:0
TAG 42:2	C_45_H_82_O_6_	736.6455	8:0/16:0/18:2	10:0/14:0/18:2	6:0/18:1/18:1
TAG 42:1	C_45_H_84_O_6_	738.6612	10:0/14:0/18:1	8:0/16:0/18:1	12:0/12:0/18:1
TAG 42:0	C_45_H_86_O_6_	740.6768	12:0/14:0/16:0	8:0/16:0/18:0	10:0/14:0/18:0
TAG 44:1	C_47_H_88_O_6_	766.6925	10:0/16:0/18:1	12:0/14:0/18:1	12:0/16:0/16:1
TAG 44:0	C_47_H_90_O_6_	768.7081	14:0/14:0/16:0	10:0/16:0/18:0	4:0/16:0/24:0
TAG 45:2	C_48_H_88_O_6_	778.6926	12:0/15:0/18:2	10:0/17:1/18:1	14:1/15:0/16:1
TAG 45:1	C_48_H_90_O_6_	780.7082	14:0/15:0/16:1	14:1/15:0/16:0	12:0/15:0/18:1
TAG 45:0	C_48_H_92_O_6_	782.7238	14:0/15:0/16:0	12:0/16:0/17:0	
TAG 46:2	C_49_H_90_O_6_	792.7081	10:0/18:1/18:1	12:0/16:1/18:1	14:0/14:1/18:1
TAG 46:1	C_49_H_92_O_6_	794.7238	12:1/16:0/18:0	14:0/16:0/16:1	12:0/16:0/18:1
TAG 46:0	C_49_H_94_O_6_	796.7394	14:0/16:0/16:0	15:0/15:0/16:0	
TAG 48:3	C_51_H_92_O_6_	818.7238	14:1/16:1/18:1	14:0/16:0/18:3	14:0/16:1/18:2
TAG 48:2	C_51_H_94_O_6_	820.7394	14:0/16:1/18:1	14:0/16:0/18:2	12:0/18:1/18:1
TAG 48:1	C_51_H_96_O_6_	822.7551	14:0/16:0/18:1	16:0/16:0/16:1	
TAG 48:0	C_51_H_98_O_6_	824.7707	14:0/16:0/18:0	16:0/16:0/16:0	15:0/16:0/17:0
TAG 49:2	C_52_H_96_O_6_	834.7551	15:0/16:0/18:2	16:0/16:1/17:1	15:0/16:1/18:1
TAG 49:1	C_52_H_98_O_6_	836.7707	15:0/16:0/18:1	16:0/16:0/17:1	
TAG 50:4	C_53_H_94_O_6_	844.7394	14:0/18:2/18:2	14:0/18:1/18:3	16:1/16:1/18:2
TAG 50:3	C_53_H_96_O_6_	846.7551	14:0/18:1/18:2	16:0/16:1/18:2	14:1/18:1/18:1
TAG 50:2	C_53_H_98_O_6_	848.7707	16:0/16:1/18:1	14:0/18:1/18:1	16:0/16:0/18:2
TAG 50:1	C_53_H_100_O_6_	850.7864	16:0/16:0/18:1	16:0/16:1/18:0	16:0/17:0/17:1
TAG 50:0	C_53_H_102_O_6_	852.8020	16:0/16:0/18:0		
TAG 51:4	C_54_H_96_O_6_	858.7551	15:0/18:2/18:2	15:0/18:1/18:3	
TAG 51:3	C_54_H_98_O_6_	860.7707	15:0/18:1/18:2	16:0/17:0/18:3	16:1/17:1/18:1
TAG 51:2	C_54_H_100_O_6_	862.7864	15:0/18:1/18:1	16:0/17:1/18:1	16:1/17:0/18:1
TAG 51:1	C_54_H_102_O_6_	864.8021	16:0/17:0/18:1	16:0/17:1/18:0	15:0/18:0/18:1
TAG 51:0	C_54_H_104_O_6_	866.8177	14:0/16:0/21:0	16:0/17:0/18:0	
TAG 52:5	C_55_H_96_O_6_	870.7551	14:0/18:1/20:4	16:0/16:1/20:4	16:1/18:2/18:2
TAG 52:4	C_55_H_98_O_6_	872.7707	16:0/18:2/18:2	16:0/18:1/18:3	16:1/18:1/18:2
TAG 52:3	C_55_H_100_O_6_	874.7864	16:0/18:1/18:2	16:1/18:1/18:1	16:0/16:0/20:3
TAG 52:2	C_55_H_102_O_6_	876.8020	16:0/18:1/18:1	17:0/17:1/18:1	
TAG 52:1	C_55_H_104_O_6_	878.8177	16:0/18:0/18:1	16:0/16:0/20:1	
TAG 52:0	C_55_H_106_O_6_	880.8333	16:0/18:0/18:0	14:0/16:0/22:0	17:0/17:0/18:0
TAG 54:5	C_57_H_100_O_6_	898.7864	18:1/18:2/18:2	18:1/18:1/18:3	
TAG 54:4	C_57_H_102_O_6_	900.8020	18:1/18:1/18:2	18:0/18:1/18:3	
TAG 54:3	C_57_H_104_O_6_	902.8177	18:0/18:1/18:2	18:0/18:0/18:3	
TAG 54:2	C_57_H_106_O_6_	904.8333	18:1/18:1/18:1	16:0/18:1/20:1	
TAG 54:1	C_57_H_108_O_6_	906.8490	16:0/16:1/22:0	14:0/16:1/24:0	
TAG 54:0	C_57_H_110_O_6_	908.8646	16:0/18:0/20:0	18:0/18:0/18:0	
TAG 56:3	C_59_H_108_O_6_	930.8490	18:0/18:0/20:3	18:0/18:3/20:0	18:1/18:1/20:1
TAG 56:2	C_59_H_110_O_6_	932.8646	16:1/18:1/22:0	18:0/18:1/20:1	18:1/18:1/20:0
TAG 56:1	C_59_H_112_O_6_	934.8803	16:0/18:1/22:0	16:0/16:1/24:0	

Even using two LC columns and a 90-min gradient elution, complete chromatographic separation of all isomer species within most TAG groups was not achieved. For example, in the case of TAG 26:0 group, among the five species identified by LipidSearch, the three main isomers were not resolved completely (Fig. S1B, Supporting Information), so reliable quantification at the species (isomer) level is not currently feasible. Consequently, our survey on TAG abundance was conducted at the group level using a short LC method, which allowed all main isomers to be eluted as a single peak and enabled also a higher throughput sample analysis (Fig. S1A, Supporting Information).

**Figure 1:**
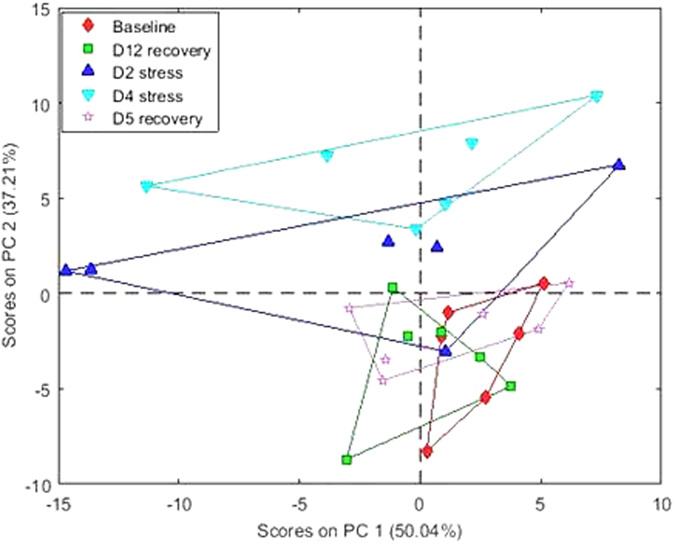
Unsupervised classification by principal component analysis of samples collected at five time points from one cohort of six experimental cows.

**Figure 2:**
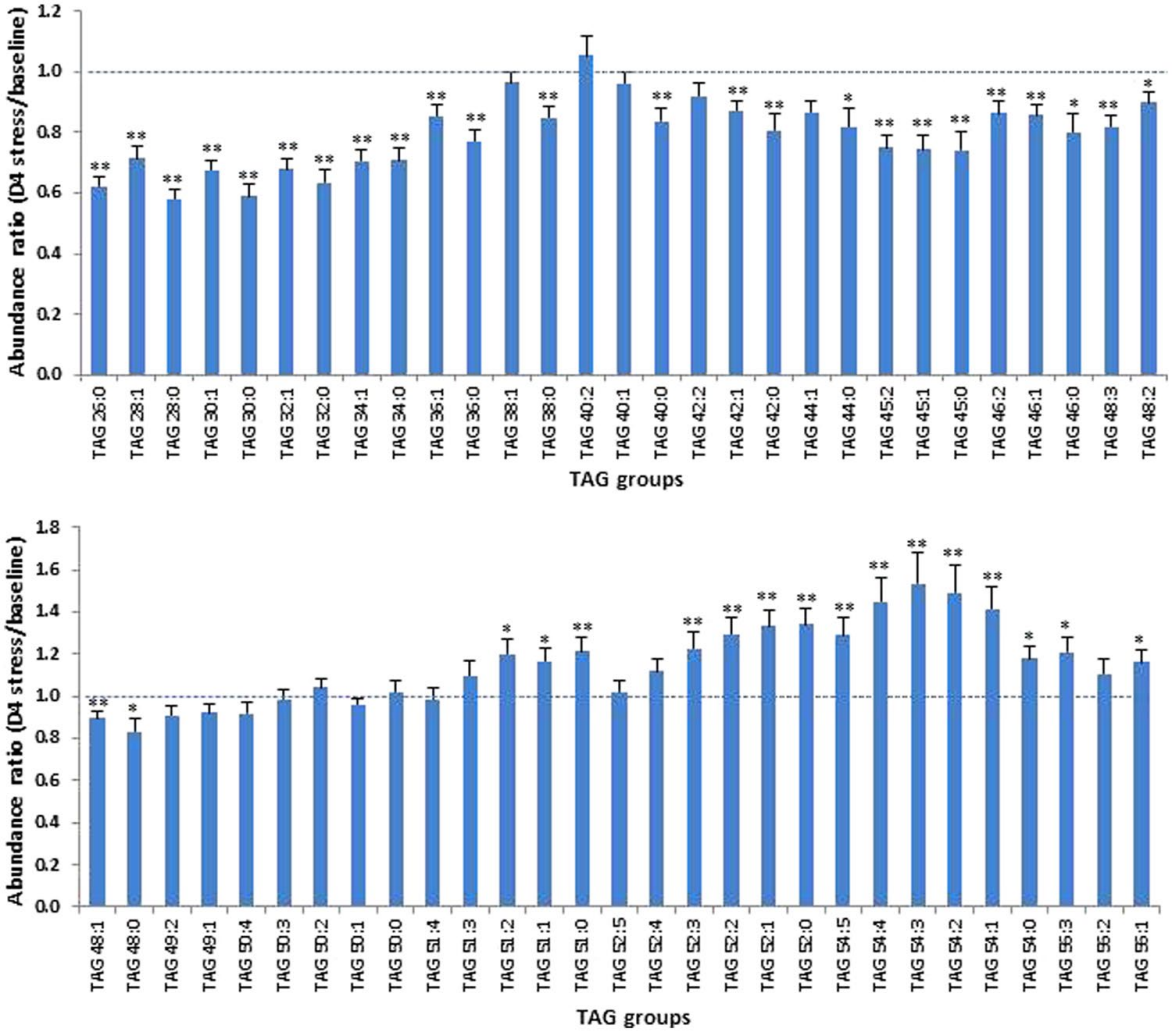
Effect of heat stress on the abundance of 58 TAG groups in milk (from afternoon milking). Each column represents the abundance ratio between D4 stress and baseline (control) samples for one TAG group. Error bars are standard error (n = 30). Statistical difference is shown by *(P < 0.05) and **(P < 0.01).

A preliminary survey using samples collected at five time points from one cohort of six cows showed that the overall TAG profile, as revealed by PCA, shifted noticeably from the baseline after 2 days’ heat challenge and a complete separation from the baseline was observed after 4 days’ heat challenge, whereas no remarkable difference in TAG profile was found between pre-heat challenge (baseline) and post-heat challenge (recovery) samples (Fig. 1). As a result, further analysis on all the 5 cohorts of 30 cows was focused only on the comparison between baseline and D4 heat challenge samples. Due to the large difference in abundance across the 58 TAG groups, the effect of heat stress on the abundance of each TAG group is presented as the abundance ratio between D4 heat stress and baseline (control) samples (Fig. 2).

It appears that the effect of heat stress on the relative abundance of TAG groups varies mostly with their CN. The heat challenge caused a significant reduction of TAG groups containing 26–48 CN regardless of the level of unsaturation, whereas it increased the level of those with 51–56 CN, irrespective of the number of BD (Fig. 2). The TAG groups showing the greatest reduction are TAG 28:0 and TAG 30:0 (by about 40% for both), and those showing the greatest increase are TAG 54:3 and TAG 54:2 (by about 50% for both). For all TAG groups with 49–50 CN, heat stress had little impact. It is interesting to note that three TAG groups containing a FA with odd number of carbons (TAG 45:2, TAG 45:1 and TAG 45:0) showed a significant reduction in heat stressed cows (Fig. 2).

At the group level, TAG 28:0 sustained the greatest reduction by heat stress, but whether all isomer species of this group are affected at the same magnitude remains a question, given that each TAG group contains on average 4–5 isomer species. The long LC separation method allowed us to resolve chromatographically 6 species from this TAG group with different FA composition, and also revealed that one of the minor species TAG 4:0/6:0/18:0 was not reduced at the same level as other species by heat stress (Fig. S2, Supporting Information).

It is well established that most of the C4:0 to C14:0 and almost half of the C16:0 FA in milk are synthesized *de novo* in the mammary gland, whereas the rest of the C16:0 and approximately all long-chain FA originate from blood lipids^24, 25^. If we classify the major FA into 3 categories, namely short chain FA (SCFA, C4-C10), medium-chain FA (MCFA, C12-C16) and long-chain FA (LCFA, ≥C18), the main isomer species of the 58 TAG groups feature a number of configurations, *i.e*. S-S-M, S-M-M, S-M-L, S-L-L, M-L-L and L-L-L (Table 1). TAG groups that showed a significant reduction after heat stress belong mainly to the first 3 types of configurations, which are composed of predominantly SCFA and MCFA, whereas those that are induced by heat stress are from the last 2 types of configurations containing mainly LCFA.

Global FA composition analysis of milk samples from three cohorts (18 animals) by GC-MS revealed that the level of C4:0-C15:0 was indeed significantly reduced, whereas that of LCFA especially C18:0, C18:1 and C18:2 significantly increased by the heat challenge (Table 2). These results are in agreement with the data reported by Lacetera *et al*.^16^ and Hammami *et al*.^12^, who described a decrease in content of SCFA and MCFA and an increase in that of LCFA from heat-stressed cows. It is interesting to mention that the ratios of C14:1 to C14:0, C16:1 to C16:0 and C18:1 to C18:0 were very close between baseline and D4 heat stress samples, implying the activity of Δ9-desaturase was not affected by heat stress.

**Table 2:** Effect of heat stress on fatty acid composition of milk fat.

Fatty acid	mg/g of milk fat	
	Baseline	D4 heat stress
C4:0	16.9	13.3*
C6:0	16.5	12.4**
C8:0	11.7	8.5**
C10:0	32.1	21.4**
C12:0	38.1	25.0**
C14:0	112.2	84.2**
C14:1	9.8	6.2**
C15:0	14.5	10.4**
C16:0	319.0	266.1*
C16:1	15.4	13.8
C17:0	7.5	9.2
C18:0	60.3	94.6**
C18:1n9c	128.4	191.6**
C18:2n6c	16.0	20.1*
C18:3n3	8.5	9.1
C20:0	2.2	2.6
C22:0	1.8	1.8
Total *de novo*	251.8	181.4**
Total C16:0+C16:1	334.4	279.9
Total preformed	271.4	324.5**

Total *de novo* = sum of C4:0 to C15:0 fatty acids; total preformed = sum of all fatty acids with more than 17 carbon atoms. Each value is the mean of 18 samples. Statistical difference is shown by *(P < 0.05) and **(P < 0.01).

As *de novo* synthesis of C4-C14 FA is from acetate and butyrate, which are generated in the rumen by fermentation of feed components^17^, heat stress-induced reduction of C4:0-C14:0 FA-rich TAG groups may partly result from lowered dry matter intake as recorded by Garner *et al*.^5^ in this same experiment, which led to suppressed ruminal fermentation and consequently reduced supply of acetate and butyrate. However, diminished anabolic activities including synthesis of fatty acids in mammary gland due to partitioning of energy in thermoregulation-related processes in response to heat stress may also be responsible^1, 4^. So the reduction of TAG groups with 26–48 CN can be attributed to the reduced synthesis of *de novo* FA under heat stress. This could also explain the reduction of TAG 45:2, TAG 45:1 and TAG 45:0 which contain C15:0, a FA thought to be synthesised by the bacteria in the rumen^26^. By contrast, half of C16:0 and all of LCFA are not synthesised in the mammary gland, but originate from dietary lipids and from lipolysis of adipose tissue TAG^25^, so the supply of these FA may not be affected or even be induced by heat stress. Consequently, LCFA-rich TAG species (>50 CN) show an increase in relative proportions.

TAG composition is known to affect the property of dairy product. For example, the spread ability of butter produced in winter is much lower than that of summer butter, due to the higher level of unsaturated FA in summer milk^27, 28^. A combination of reduced proportion of SCFA-rich TAG and an increased proportion of LCFA-rich TAG is expected to increase the viscosity and melting point of milk fat produced by heat stressed cows, which may have impact on the processing and property of dairy products.

The 5 cohorts of 30 cows used in our study were equally distributed for heat sensitivity, *i.e*. 15 were heat tolerant (HT) and 15 were heat susceptible (HS). When the TAG profile of these two types of cows was compared, no significant difference was found for any of the TAG groups in D4 stress samples, nor in baseline samples (results not shown). So it appears that the heat sensitivity trait in dairy cows is not associated with the TAG composition of milk fat.

### Effect of heat stress on polar lipid content

Eight classes of polar lipids were surveyed in this study. Over 100 species were identified by LipidSearch based on MS/MS spectrum, of which 58 most abundant species were selected for relative quantification; the formula, accurate mass and FA composition of these species are given in Table 3. The absolute content of the 8 classes of polar lipids in raw milk was described in our previous report^29^. In this study, only relative quantification was performed at the species level (based on peak area of each species) and at the class level (based on the sum of all species within the same class). To illustrate the effect of heat stress on the abundance of each polar lipid class, the abundance ratio between D4 heat stress and baseline samples is again presented.

**Table 3:** List of polar lipid species surveyed.

PL class	Fatty acids	Ion detected	Formula	Calculated m/z
PS	(16:0/18:2)	M + H	C_40_H_75_O_10_N_1_P_1_	760.5123
PS	(16:0/18:1)	M + H	C_40_H_77_O_10_N_1_P_1_	762.5280
PS	(18:1/18:2)	M + H	C_42_H_77_O_10_N_1_P_1_	786.5280
PS	(18:0/18:2)	M + H	C_42_H_79_O_10_N_1_P_1_	788.5436
PS	(18:0/18:1)	M + H	C_42_H_81_O_10_N_1_P_1_	790.5593
PS	(18:1/20:4)	M + H	C_44_H_77_O_10_N_1_P_1_	810.5285
PS	(18:0/20:4)	M + H	C_44_H_79_O_10_N_1_P_1_	812.5436
PS	(18:0/22:5)	M + H	C_46_H_81_O_10_N_1_ P_1_	838.5593
PE	(18:1/14:0)	M + H	C_37_H_73_O_8_N_1_P_1_	690.5068
PE	(16:0/18:2)	M + H	C_39_H_75_O_8_N_1_P_1_	716.5225
PE	(16:0/18:1)	M + H	C_39_H_77_O_8_N_1_P_1_	718.5381
PE	(18:1/18:3)	M + H	C_41_H_75_O_8_N_1_P_1_	740.5225
PE	(18:1/18:2)	M + H	C_41_H_77_O_8_N_1_P_1_	742.5381
PE	(18:1/18:1)	M + H	C_41_H_79_O_8_N_1_P_1_	744.5538
PI	(16:0/18:1)	M − H	C_43_H_81_O_13_P_1_	835.5337
PI	(18:1/18:1)	M − H	C_45_H_83_O_13_P_1_	861.5493
PI	(18:1/18:0)	M − H	C_45_H_85_O_13_P_1_	963.5650
PI	(18:0/20:5)	M − H	C_47_H_81_O_13_P_1_	883.5337
PI	(18:0/20:4)	M − H	C_47_H_83_O_13_P_1_	885.5493
PI	(18:0/20:3)	M − H	C_47_H_85_O_13_P_1_	887.5650
PC	(14:0/14:0)	M + H	C_36_H_73_O_8_N_1_P_1_	678.5068
PC	(16:0/14:0)	M + H	C_38_H_77_O_8_N_1_P_1_	706.5381
PC	(15:0/16:0)	M + H	C_39_H_79_O_8_N_1_P_1_	720.5538
PC	(16:0/16:1)	M + H	C_40_H_79_O_8_N_1_P_1_	732.5538
PC	(16:0/16:0)	M + H	C_40_H_81_O_8_N_1_P_1_	734.5694
PC	(16:0/18:3)	M + H	C_42_H_79_O_8_N_1_P_1_	756.5538
PC	(16:0/18:2)	M + H	C_42_H_81_O_8_N_1_P_1_	758.5694
PC	(16:0/18:1)	M + H	C_42_H_83_O_8_N_1_P_1_	760.5851
PC	(18:2/18:2)	M + H	C_44_H_81_O_8_N_1_P_1_	782.5694
PC	(18:1/18:2)	M + H	C_44_H_83_O_8_N_1_P_1_	784.5851
PC	(18:1/18:1)	M + H	C_44_H_85_O_8_N_1_P_1_	786.6007
LPC	(16:0)	M + H	C_24_H_51_O_7_N_1_P_1_	496.3398
LPC	(18:3)	M + H	C_26_H_49_O_7_N_1_P_1_	518.3241
LPC	(18:2)	M + H	C_26_H_51_O_7_N_1_P_1_	520.3398
LPC	(18:1)	M + H	C_26_H_53_O_7_N_1_P_1_	522.3554
LPC	(18:0)	M + H	C_26_H_55_O_7_N_1_P_1_	524.3716
SM	(d16:1/16:0)	M + H	C_37_H_76_ O_6_N_2_P_1_	675.5436
SM	(d16:0/17:1)	M + H	C_38_H_78_O_6_N_2_P_1_	689.5592
SM	(d18:1/16:0)	M + H	C_39_H_80_O_6_N_2_P_1_	703.5749
SM	(d16:1/22:0)	M + H	C_43_H_88_O_6_N_2_P_1_	759.6375
SM	(d16:1/23:0)	M + H	C_44_H_90_O_6_N_2_P_1_	773.6531
SM	(d18:1/22:1)	M + H	C_45_H_90_O_6_N_2_P_1_	785.6537
SM	(d18:1/22:0)	M + H	C_45_H_92_O_6_N_2_P_1_	787.6688
SM	(d18:1/23:1)	M + H	C_46_H_92_O_6_N_2_P_1_	799.6688
SM	(d18:1/23:0)	M + H	C_46_H_94_O_6_N_2_P_1_	801.6844
SM	(d18:1/24:1)	M + H	C_47_H_94_O_6_N_2_P_1_	813.6844
SM	(d18:1/24:0)	M + H	C_47_H_96_O_6_N_2_P_1_	815.7001
LacCer	(d18:1/16:0)	M + H	C_46_H_87_O_13_N_1_	862.6256
LacCer	(d18:1/20:0)	M + H	C_50_H_95_O_13_N_1_	918.6882
LacCer	(d18:0/20:0)	M + H	C_50_H_97_O_13_N_1_	920.7038
LacCer	(d16:1/23:0)	M + H	C_51_H_97_O_13_N_1_	932.7038
LacCer	(d16:0/23:0)	M + H	C_51_H_99_O_13_N_1_	934.7195
LacCer	(d18:1/22:0)	M + H	C_52_H_99_O_13_ N_1_	946.7195
LacCer	(d18:1/23:0)	M + H	C_53_H_101_O_13_N_1_	960.7351
LacCer	(d18:1/24:0)	M + H	C_54_H_103_O_13_N_1_	974.7508
GluCer	(d18:1/16:0)	M + H	C_40_H_77_O_8_N_1_	700.5727
GluCer	(d18:1/22:0)	M + H	C_46_H_89_O_8_N_1_	784.6666
GluCer	(d18:1/24:0)	M + H	C_48_H_93_O_8_N_1_	812.6979

**Figure 3:**
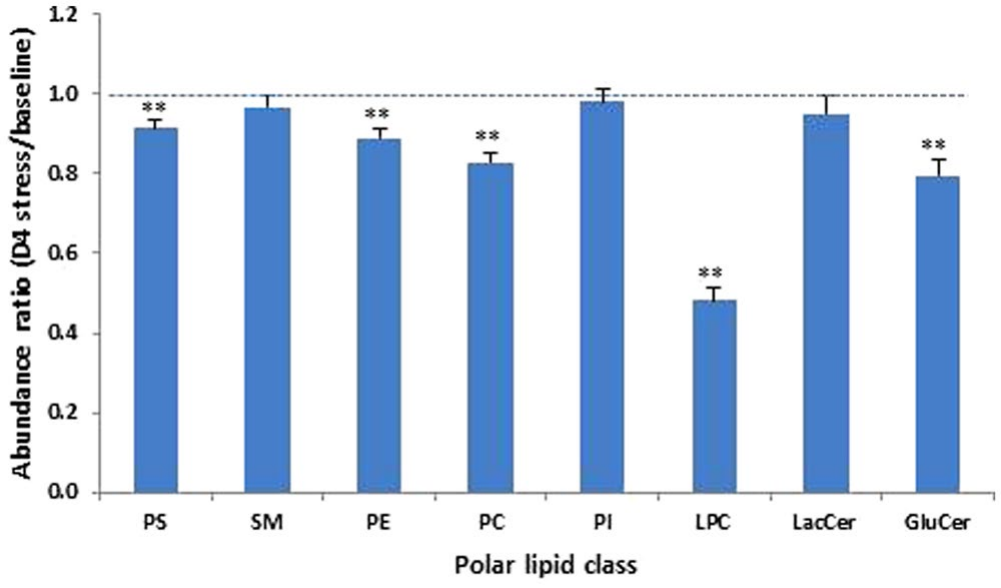
Effect of heat stress on the abundance of eight polar lipid classes in milk (from afternoon milking). Each column represents the abundance ratio between D4 stress and baseline (control) samples for one polar lipid class. Error bars are standard error (n = 30). Statistical difference is shown by **(P < 0.01).

At the class level, a 4-day heat challenge had no significant influence on the abundance of PI, SM and LacCer, but reduced that of PS, PE, PC, LPC and GluCer (Fig. 3). While only a slight decrease was observed for PS (9%) and PE (11%), a substantial reduction was recorded for PC (17%) and GluCer (21%). However, the most remarkable change in polar lipid content brought about by heat stress was detected with LPC, for which a dramatic reduction (52%) was observed after 4 days’ heat stress treatment (Fig. 3). It is worth mentioning that the abundance of all the five species within this class was dramatically reduced (results not shown).

**Figure 4:**
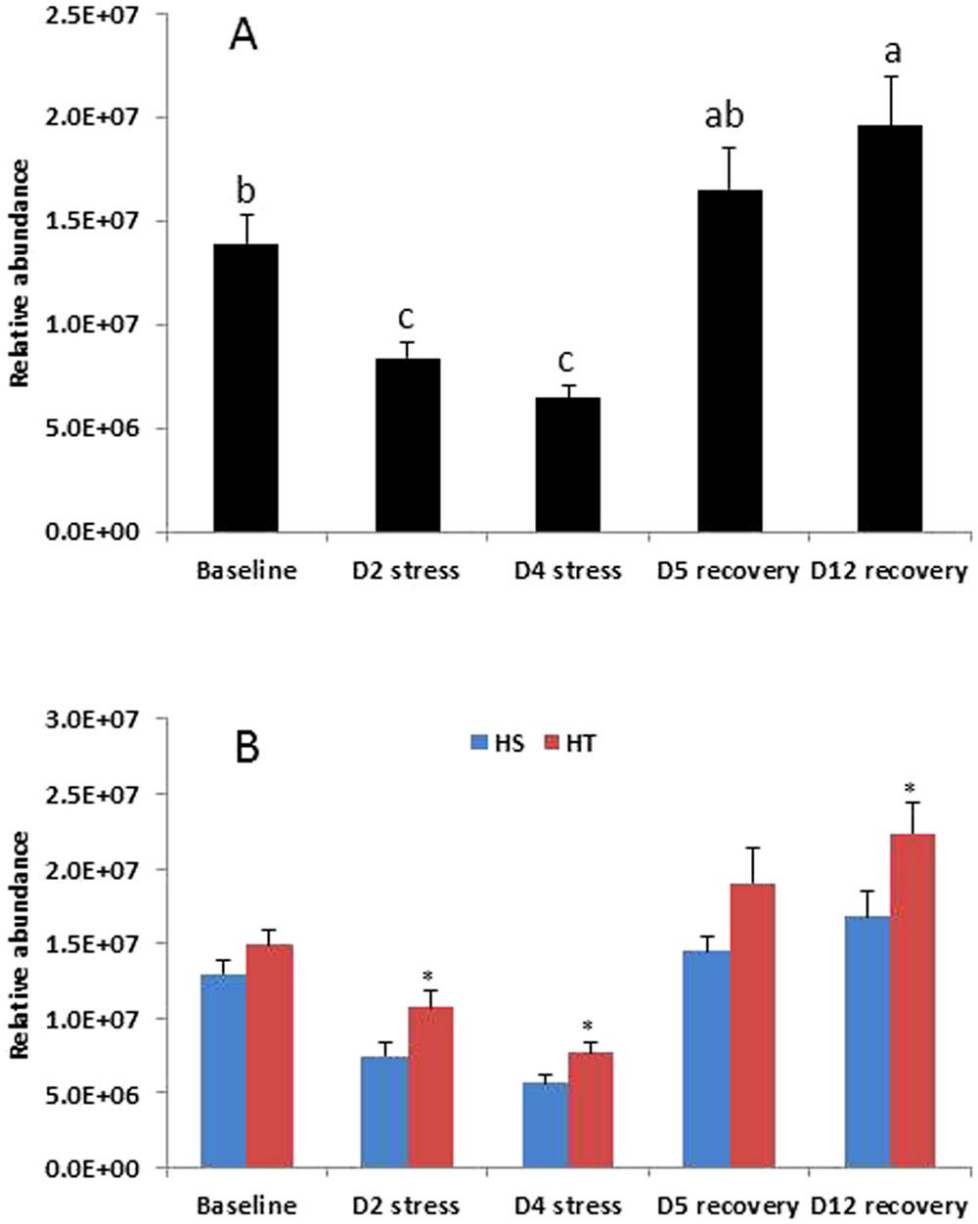
Change in LPC abundance in milk (from afternoon milking) during the heat stress experiment. (**A**) Comparison of milk LPC abundance across different time points. Each column represents the mean abundance of all 30 cows. Error bars are standard error (n = 30). Columns with different letters are significantly different (P < 0.05). (**B**) Comparison of milk LPC abundance between HT and HS cows at different time points. Each column represents the mean abundance of 15 HT or HS cows. Error bars are standard error (n = 15). Statistical difference for each column pair is shown by *(P < 0.05).

A detailed survey of LPC level was conducted for all the 5 time points of the experiment. Indeed, the level of LPC was significantly decreased (by 34%) after 2 days’ heat challenge, which dropped further after 4 days’ treatment and then rose to a normal level after 5 days’ recovery (Fig. 4A). In addition to the dramatic decrease after 2 days’ heat treatment, LPC displayed a differential response between HT and HS cows in the event of heat stress. Figure 4B shows that the LPC level is not significantly different between HT and HS cows at the baseline point, but the heat induced reduction of this polar lipid class is much less with HT as compared to HS animals regardless of the duration of heat challenge. This LPC changing pattern is similar to the results reported by Garner *et al*.^5^, who observed a lower body temperature, a higher dry matter intake and a higher milk yield of HT cows as compared to HS cows during the heat challenge. Consequently, LPC can be regarded as a heat stress biomarker for dairy cattle. In this regard, it would be interesting to investigate the LPC level after a short period (for example, a few hours) of heat stress to further validate this finding.

The effect of heat stress on polar lipid composition has not been extensively investigated. A recent study by Tian *et al*.^30^ using untargeted metabolic profiling approach identified a large number of potential heat stress biomarkers including some lipid species, but one single LPC species included in that list was found to be induced in heat stressed cows. This discrepancy may be attributable to different experimental designs, ambient conditions (baseline level) *versus* controlled environment (heat stress) in our experiment as compared to summer samples *versus* spring samples in their study.

Compared with TAG, polar lipids are minor component in milk fat (<1%). However, our study revealed that one polar lipid class LPC is much more sensitive to heat stress than TAG as a whole, since maximum reduction observed on TAG 28:0 and TAG 30:0 was 40% after a 4-day heat challenge, as compared to over 50% reduction in the case of LPC. While the heat-induced decrease of some TAG groups appears to be caused by a decline in *de novo* FA, this same mechanism solely cannot explain the reduction of LPC which contains only C16 and LCFA in the five species surveyed (Table 3). Some up-stream steps in the biosynthesis pathways, such as the formation of choline/phosphocholine may be inhibited under heat stress. Clearly, further investigation is needed to understand the underlying mechanisms. It is also worth noting that the simulated heat challenge in the current experiment is of moderate magnitude (maximum temperature 33 °C and THI up to 84). How milk lipid composition changes in more severe heat stress conditions remains to be determined.

We have found that 5 out of the 8 polar lipid classes were affected by heat stress, but the overall level of polar lipids is not expected to be remarkably reduced, since LPC and GluCer are minor classes, whereas PS, PE and PC were only slightly and moderately suppressed. In addition, our previous study demonstrated that the major classes of polar lipids (PC, PE, PS, PI and SM) in milk were correlated with each other^29^. Such an inter-class correlation was not affected by heat stress. As an example, a rather similar correlation level was observed between PE and PC in D4 stress samples as compared to the baseline samples (Fig. S3, Supporting Information).

It should be pointed out that our simulated heat stress in a controlled climate chamber can be considered as an acute heat stress, because it was conducted in late winter/early spring with an ambient temperature below 20 °C and THI below 70. The effect of such an acute heat stress on animal metabolism may be different from the seasonal long term heat stress experienced by animals in summer, as in the latter case heat load imposed on dairy cows is gradual but prolonged. A parallel study on milk lipid composition as influenced by a chronic heat stress is still lacking.

## Conclusion

In conclusion, heat stress could alter the TAG profile of milk, which is featured by a decrease of TAG groups containing predominantly SCFA to MCFA and a concomitant increase of those containing mostly LCFA. The heat challenge also significantly reduced the level of some polar lipid classes especially LPC, which appears to be a lipid marker for heat stress in dairy cattle. While the TAG profile was modified in a similar manner for both HT and HS cows, a higher level of LPC was detected for HT cows as compared to HS cows during the heat stress.

## Materials and Methods

### Cows and experimental design

The experiment received animal ethics approval from the Agricultural Research and Extension Animal Ethics Committee of the Department of Economic Development, Jobs, Transports and Resources, Victoria, Australia. All methods were performed by approved staff members in accordance with the relevant standard operating procedures approved by the above mentioned ethics committee. Detailed information on animals and experimental design were described by Garner *et al*.^5^. In brief, 48 primiparous Holstein-Friesian cows were used in this experiment; 24 cows were genomically selected to be heat tolerant (HT) and 24 cows heat susceptible (HS). At the beginning of the experiment, mean DIM was 67 for HT cows and 68 for HS cows and mean body weight was 477 kg for HT cows and 488 kg for HS cows. The total duration of the experiment was 25 consecutive days including a 7 day baseline measurement period outside in ambient conditions, a 4 day heat challenge in controlled-climate chambers, and a 14 day recovery period outside in ambient conditions.

The 48 cows were introduced to controlled climate chambers in 8 cohorts. Temperature and relative humidity inside the controlled-climate chambers was varied to mimic the diurnal patterns in heat load imposed on dairy cows that occur during heat-wave events in southern Australia. The conditions in the controlled-climate chambers were designed to remain above THI 72 and not exceed THI 84 to impose a moderate level of heat stress. The climatic conditions programmed into the control system were 25 °C and 60% RH (THI 74) between 6 pm and 6 am, 30 °C and 50% RH (THI 80) between 6 am and 12 noon, and 33 °C and 50% RH (THI 84) between 12 noon and 6 pm. The 12 hour light and 12 hour dark cycle was controlled manually. The animal diet during the experimental period was described in Garner *et al*.^5^.

Milk samples were collected at five time points during the experiment period, on day 3 of the baseline period (baseline), on day 2 and 4 of the heat challenge in controlled-climate chambers (D2 stress and D4 stress), and at day 5 and 12 of the recovery period (D5 recovery and D12 recovery). Cows were milked twice daily (6:00 am and 3:00 pm) and the samples from afternoon milking were analysed for lipid composition. The milk samples were transported to the laboratory on ice and stored in −80 °C. A subset of samples from 5 randomly selected cohorts totalling 30 cows (15 HT and 15 HS) were analysed to determine the effect of heat stress on lipid composition of raw milk.

### Chemicals and reagents

One TAG species (TAG tri-20:1) used as internal standard for TAG analysis was purchased from Sigma-Aldrich. One PS species (PS 17:0/17:0) used as internal standard for polar lipid analysis was purchased from Avanti Lipids.

Solvents used for lipid extraction and mobile phase preparation were of chromatographic grade and were from Merck (methanol, butanol and acetonitrile) and Sigma-Aldrich (chloroform and isopropanol). Ammonium formate, used as mobile phase additive, was of analytical grade (Sigma-Aldrich).

### Lipid extraction from milk for LC-MS analysis

Raw milk samples were diluted by adding 2 volumes of Milli-Q water. Lipid extraction from the diluted milk samples was conducted using the one phase method recently developed^31^. Briefly, one mL of lipid extraction mix (butanol/methanol/chloroform at a 3:5:4 ratio) was added to 100 µL of diluted milk. The mixture was shaken by vortex for 20 s, sonicated for 20 min and then centrifuged for 15 min (15000 *g*). The supernatant was transferred to an injection vial and analysed directly by LC-MS.

### LC-MS method

Chromatographic separation for TAG identification was achieved using two Poroshell 120 EC-C18 columns (150 × 4.6 mm, 2.7 µm, Agilent Technologies) connected in series on an Agilent 1290 Infinity HPLC system. The column compartment was maintained at 40 °C and the auto-sampler at 12 °C. The mobile phase was composed of acetonitrile/water (60:40, v/v) containing 10 mM ammonium formate (A) and acetonitrile/isopropanol (10:90, v/v) containing 10 mM ammonium formate (B). The flow rate was 0.5 mL/min with a gradient elution of 60 to 100% B over 90 min. The injection volume was 4 µL.

Chromatographic separation for TAG quantification was conducted using an Acquity UPLC HSS T3 column (100 × 2.1 mm, 1.8 µm, Waters) on the same Agilent HPLC system. The column compartment was maintained at 50 °C and the auto-sampler at 12 °C. The mobile phase was composed of acetonitrile/water (60:40, v/v) containing 10 mM ammonium formate (A) and acetonitrile/isopropanol (10:90, v/v) containing 10 mM ammonium formate (B). The flow rate was 0.28 mL/min with a gradient elution of 20 to 100% B over 20 min. The injection volume was 2 µL.

Chromatographic separation for polar lipid identification and quantification was performed using a Luna HILIC column (250 × 4.6 mm, 5 µm, Waters) on the aforementioned HPLC system. The column compartment was maintained at 30 °C and the auto-sampler at 12 °C. The mobile phase was composed of 5 mM aqueous ammonium formate (A) and acetonitrile +0.1% formic acid (B). The flow rate was 0.6 mL/min with a gradient elution of 2 to 21% A over 25 min. The injection volume was 5 µL.

The detection of lipids was by LTQ-Orbitrap Elite mass spectrometer (Thermo Scientific) operated in electrospray ionization positive (for analysis of TAG and most polar lipid classes) or negative (for analysis of PI) Fourier transform mode. The resolution was set to 60,000 for both positive and negative modes. Identification of lipid species present in milk was performed based on accurate mass of parent ions (±5 ppm) and product ions (±10 ppm) as well as top5 MS/MS spectra (CE 35) using LipidSearch software (version 4.1, Thermo Scientific)^32^, followed by manual verification. Selected lipid species was quantified at a relative scale using peak area of parent ions after normalization by the internal standard.

### Fatty acid profiling by GC-MS

For FA composition analysis of milk fat, total lipid was extracted by the method of Bligh and Dyer^33^. After removal of chloroform under a stream of nitrogen, the transesterification of the extracted lipids was carried out by adding acidic methanol (6% H_2_SO_4_) and heating at 80 °C for 3 h^34^. The released FA methyl esters (FAMEs) were extracted by hexane and analysed by GC-MS.

The separation of FAMEs was achieved by a BPX-70 column (50 m × 0.22 mm ID, 0.25 µm film thickness, SGE Analytical Science) with a constant flow of 1.0 mL/min helium as carrier gas and the following oven temperature program: 120 °C to 245 °C ramping at 3 °C/min, with a total run time of 42 min. The injection volume was 1 µL in split mode (1:40). The detection was by an Agilent 7000 GC/MS Triple Quad with the following settings: scanning mass range of 40–550 amu, transfer line temperature of 240 °C, source temperature of 280 °C, and quad temperature of 150 °C. A standard mix (C4-C24, Supelco) containing 37 FAMEs was used to provide standard curve for quantification.

### Statistical analysis of data

All lipid content data were subjected to ANOVA (XLSTAT, Microsoft Excel); where significant differences were found between treatments, a Tukey’s HSD test was conducted for pairwise comparisons. Principal component analysis (PCA) for unsupervised classification of samples was performed with MATLAB R2014a (MathWorks, Natick, MA) utilising PLS Toolbox (Eigenvector Research, Manson, WA).

## Electronic supplementary material

Supplementary Information: Heat Stress in Dairy Cattle Alters Lipid Composition of Milk
