# Supplementary material for: Heat Stress in Dairy Cattle Alters Lipid Composition of Milk
Source: Sci Rep. 2017 Apr 19;7:961. doi: 10.1038/s41598-017-01120-9 (PMC5430412; doi:10.1038/s41598-017-01120-9)
Supplement: Supplementary file 1 — Supplementary Information: Heat Stress in Dairy Cattle Alters Lipid Composition of Milk [file 41598_2017_1120_MOESM1_ESM.pdf]

Supplementary information:

Heat stress in dairy cattle alters lipid composition of milk

Z. Liu[1*], V. Ezernieks[1], J. Wang[1], N. Wanni Arachchillage[1], J.B. Garner[2], W.J. Wales[2], B.G. Cocks[1,3] and S. Rochfort[1,3]

[1]Biosciences Research, Agriculture Victoria, AgriBio, 5 Ring Road, Bundoora, Victoria 3083, Australia

[2]Farming Systems Research, Agriculture Victoria, Ellinbank Centre, 1301 Hazeldean Rd, Ellinbank, Victoria 3821, Australia

[3]School of Applied Systems Biology, La Trobe University, Bundoora , Victoria 3083, Australia

*Corresponding author: Zhiqian.liu@ecodev.vic.gov.au

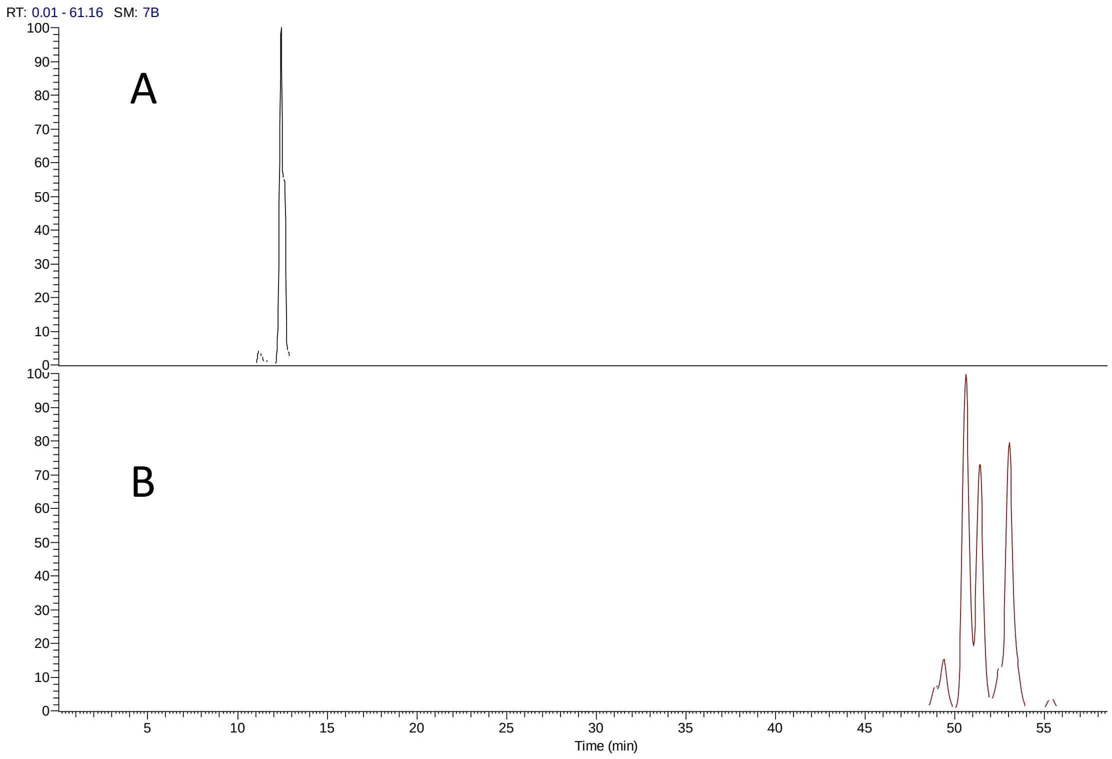

Fig. S-1: LC-MS profile of TAG 26:0 group from a baseline sample. A: short LC run for quantification at the group level; B: long LC separation for isomer species identification.

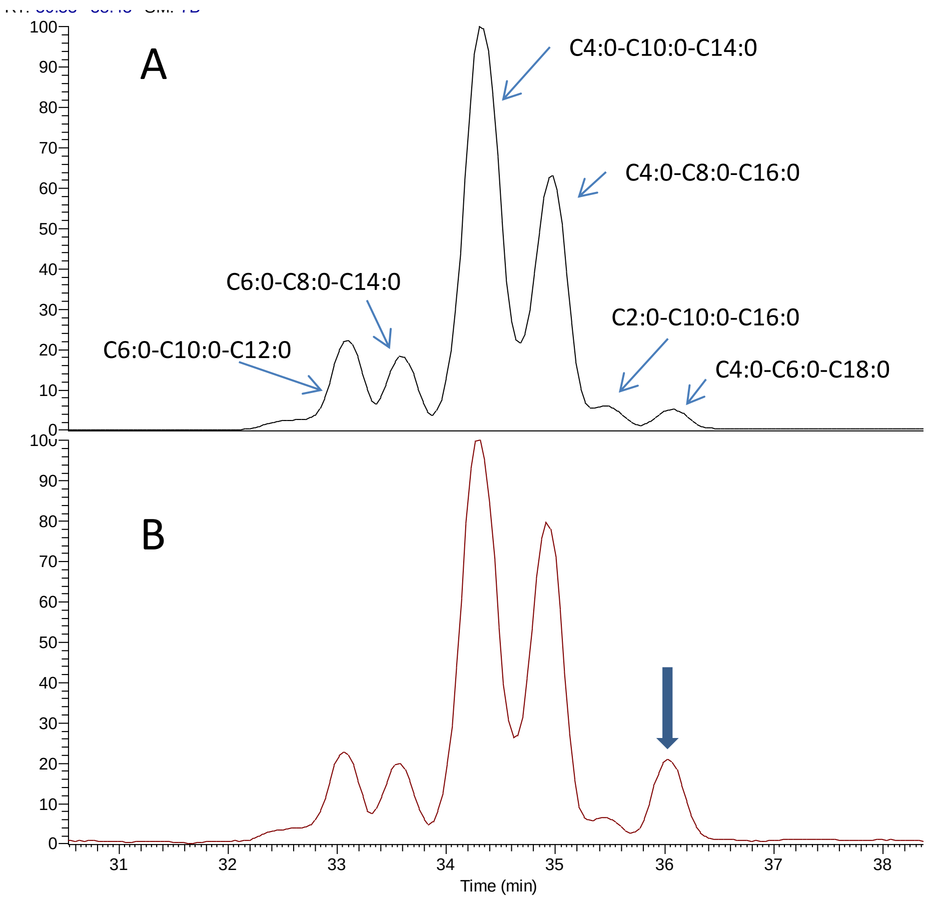

Fig. S-2: Separation of TAG 28:0 isomers by two RP columns connected in series and a long elution gradient. A: distribution of 6 isomers of a typical baseline sample; B: distribution of 6 isomers of a typical D4 stress sample (arrow indicates the isomer with altered proportion).

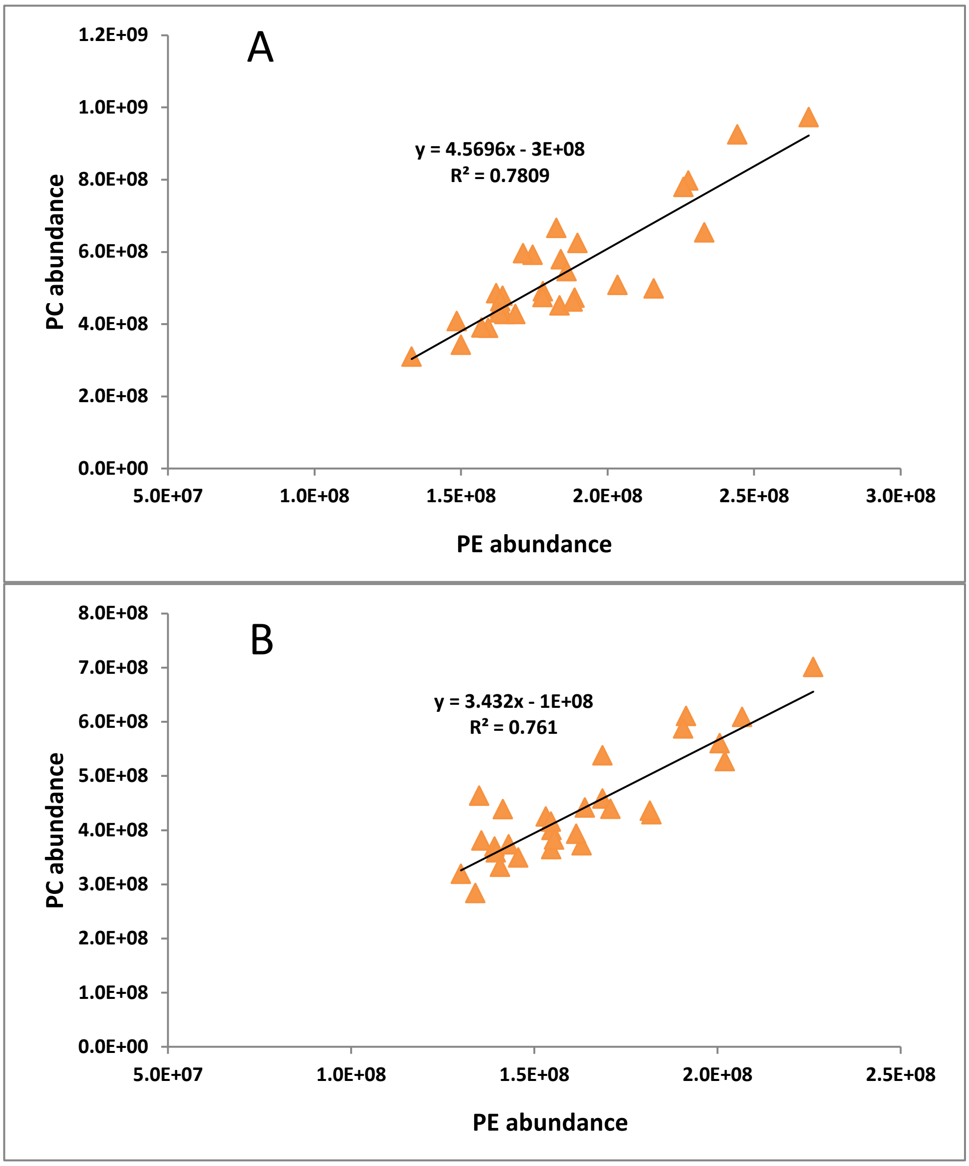

Fig. S-3: Correlation between PE and PC in milk samples collected before (A) and after a 4-day heat challenge (B).
